# Duplication and Nuclear Envelope Insertion of the Yeast Microtubule Organizing Centre, the Spindle Pole Body

**DOI:** 10.3390/cells7050042

**Published:** 2018-05-10

**Authors:** Diana Rüthnick, Elmar Schiebel

**Affiliations:** Zentrum für Molekulare Biologie der Universität Heidelberg, DKFZ-ZMBH Allianz, 69120 Heidelberg, Germany; d.ruethnick@zmbh.uni-heidelberg.de

**Keywords:** spindle pole body (SPB), nuclear pore complex (NPC), nuclear envelope (NE) insertion

## Abstract

The main microtubule organizing centre in the unicellular model organisms *Saccharomyces cerevisiae* and *Schizosaccharomyces pompe* is the spindle pole body (SPB). The SPB is a multilayer structure, which duplicates exactly once per cell cycle. Unlike higher eukaryotic cells, both yeast model organisms undergo mitosis without breakdown of the nuclear envelope (NE), a so-called closed mitosis. Therefore, in order to simultaneously nucleate nuclear and cytoplasmic MTs, it is vital to embed the SPB into the NE at least during mitosis, similarly to the nuclear pore complex (NPC). This review aims to embrace the current knowledge of the SPB duplication cycle with special emphasis on the critical step of the insertion of the new SPB into the NE.

## 1. Introduction

Fungi and animal cells contain large proteinaceous structures, collectively termed microtubule organizing centres (MTOC), that nucleate microtubules (MTs) in interphase and mitosis. In higher eukaryotes, it is mainly the centrosome that provides micro organizing functions. In fungi, it is the spindle pole body (SPB) that confers this activity. Besides the ability to assemble microtubules from tubulin subunits, centrosomes and SPBs have the remarkable ability to duplicate just once per cell cycle, similar to DNA [1,2]. The older mother structure functions as a seeding point for the assembly of the younger daughter [3,4]. The SPB is essential for viability of fungi (*Saccharomyces cerevisiae, Schizosaccharomyces pombe, Aspergillus nidulans*), because it is crucial for mitotic spindle assembly. Genes that function in centrosome duplication in human cells are also essential for viability. The reason for this is, however, is different to that of fungi. Human cells lacking centrosomes can assemble a bipolar mitotic spindle because of alternative, centrosome-independent microtubule nucleation pathways. These allow the oocytes in mammals to assemble spindles, despite the lack of centrosomes [5,6]. However, mitotic spindle assembly in the absence of centrosomes is relatively slow and error-prone. It therefore triggers a p53-dependent response that arrests mitotic cells in the subsequent G1 phase of the cell cycle [7,8]. Consequently, copy number alterations of centrosomes are tolerated in p53-cells, despite their negative impact on chromosome segregation efficiency. In fact, cancer cells that are mostly devoid of p53 activity frequently show centrosome aberrations [9,10]. There is a longstanding debate over whether they are the cause, or a consequence, of cell transformation. However, recent studies suggest that centrosome overamplification can be a cause for tumour formation in mice [11]. In addition, centrioles, which are substructures of centrosomes, function as basal bodies in the assembly of cilia. Cilia have essential functions during development, in signalling pathways and tissue homeostasis, and their loss leads to early embryonic lethality. Diseases caused by malfunction of cilia are collectively named ciliopathies [12,13].

The centrosome consists of centrioles surrounded by a cloud of proteins, the pericentriolar material. The centrioles that have a diameter of 250 nm and a length of 500 nm are microtubule-based structures containing nine triplets of microtubules [3]. Overall, it was estimated that centrosomes consist of several hundred proteins [14]. The SPB of yeast *Saccaromyces cerevisisae* (ScSPB) is the best-understood MTOC. It is a disc-like, multi-layered assembly of only 18 proteins that in haploid cells has a diameter of 80 nm in G1, and grows to 110 nm in mitosis. The width of the ScSPB is constantly 150 nm [15]. The ScSPB is embedded in the nuclear envelope (NE) throughout the cell cycle [16,17]. This embedding is a reflection of the closed mitosis of yeast *Saccharomyces cerevisisae*, and enables the ScSPB to simultaneously organize nuclear and cytoplasmic (also named astral) microtubules with functions in chromosome segregation and spindle positioning respectively. In contrast, the SPB of *Schizosaccharomyces pompe* (SpSPB) is excluded from the NE throughout interphase; it inserts prior to mitosis, and extrudes again as the cell cycle proceeds [18].

Although centrosomes and SPBs are not identical in their overall structure, shape and appearance, both MTOCs share components with functions in duplication and microtubule organization. Among these (compared and listed in [19]) is the conserved family of centrin proteins. The *S. cerevisiae* centrin gene *CDC31* (cell division cycle 31) was originally identified in a genetic screen for cell cycle genes [20]. Later it turned out to have an essential function in SPB duplication whose failure arrests cells in mitosis because of spindle assembly checkpoint (SAC) activation [21,22,23]. Mammalian centrins are a paralogues family of centriolar proteins that have been implemented in centriole biogenesis [24,25,26]. Cdc31/centrin proteins interact with the centrin binding protein Sfi1, which is present in SPBs and human centrosomes. *S. cerevisiae* and *S. pombe* Sfi1 are important for the initiation of the yeast SPB duplication process [27,28]. Interestingly, human POC5 was identified as an additional centrin-binding protein inside centrioles, that plays an important role in centriole elongation [29]. SPBs and centrosomes nucleate MTs via gamma-tubulin complexes [30,31], which are anchored to these MTOCs by binding to adapter proteins, such as Spc110_ScSPB_/Pcp1_SpSPB_/Pericentrin_centrosome_ and Spc72_ScSPB_/Mto1/2_SpSPB_/CDK5RAP2_centrosome_ [19].

These analogies qualify the SPB as a model for the cell cycle dependent duplication of a MTOC. The ease with which the yeast genome may be manipulated further allows fast analysis of gene-deletions, gene-tagging, or the introduction of mutations. Additionally, studying the insertion process of the SPB into the nuclear envelope (NE), which occurs during or after duplication, provides a deeper understanding of how large proteinaceous structures, e.g., the nuclear pore complex (NPC), become inserted into the double-lipid bilayer of the NE.

## 2. Duplication Cycle of the *S. cerevisiae* SPB

**Figure 1:**
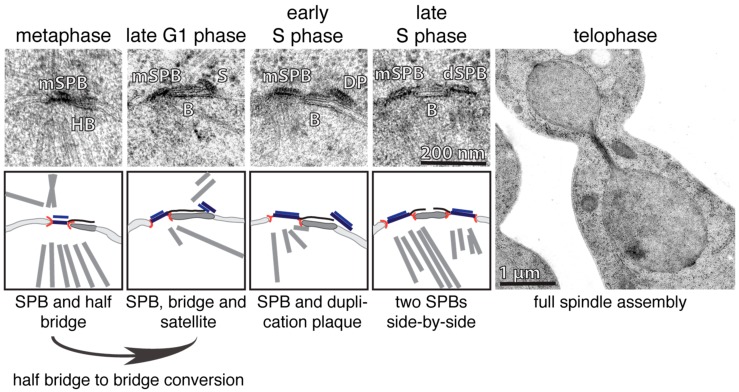
The duplication cycle of the ScSPB. The individual steps of the ScSPB duplication process are illustrated by EM micrographs (**top**) and corresponding cartoons (**bottom**). mSPB, mother SPB; HB, half bridge; B, bridge; S, satellite; DP, duplication plaque; dSPB, daughter SPB.

Just like DNA, the SPB and centrosome also have to be duplicated exactly once each cell cycle. This process was initially characterized for yeast and higher eukaryotes by electron microscopy (EM) [17,18,32,33,34,35]. EM analysis revealed that the SPB of *S. cerevisiae* (ScSPB) has a cylindrical structure that is composed of three clearly visible layers: the outer, central and inner plaque. The outer and inner plaques face the cytoplasm and nucleoplasm respectively. The central plaque is the layer that embeds the SPB into the NE [15,17,34]. Additionally, there is a one-sided extension of the central plaque covering the cytoplasmic and nuclear side of the NE, which was named the half-bridge [34] (Figure 1; metaphase). One important structural component of the half-bridge is the protein Sfi1, which forms a complex with Cdc31 [27,36]. Sfi1 is an elongated molecule that spans the entire length of the half bridge [27,36]. With increasing activity of the phosphatase Cdc14 at anaphase onset, Sfi1 becomes dephosphorylated, which in turn allows the elongation of the half-bridge into the bridge by the anti-parallel addition of Sfi1-molecules to the already existing Sfi1 layer in the half bridge [36,37,38]. In early G1-phase, the satellite, a SPB-precursor, forms at the cytoplasmic side at the distal end of the bridge [34,35]. The satellite has an ellipsoidal shape, and stands relative to its length axis upright on the bridge/NE [39,40] (Figure 1; late G1). By passing the START point of the cell cycle, the satellite grows in size, into the so-called duplication plaque (DP), which becomes inserted into the NE simultaneously with its extension. The insertion process is accompanied by a change in the orientation of the satellite. The Spc42 layer in the satellite changes from an upright to a parallel orientation in respect to the NE [40] (Figure 1; S-phase, left). This reorientation is probably an important regulatory step that prevents premature satellite insertion into the NE. The DP insertion into the NE enables nuclear components of the SPB, such as the protein Spc110, to bind to the SPB from within the nucleus, making SPB insertion irreversible. S phase cells carry two functional, MT-nucleating, side-by-side SPBs embedded in the NE (Figure 1; S-phase; right). The mother and daughter SPBs (dSPBs) are still connected via the bridge structure that is composed by two anti-parallel Sfi1 arrays that interdigitate in the bridge centre via overlapping C-termini [36,41]. To allow the formation of a bipolar spindle, the bridge has to be severed in its centre. This process is regulated through the phosphorylation of Sfi1 by cyclin dependent kinase 1 (Cdk1) [37,38]. Thus, SPB duplication is restricted to one event per cell cycle by the antagonizing functions and oscillating appearance of the kinase Cdk1 and the phosphatase Cdc14 (reviewed in [42]). This regulation exposes free Sfi1 N-termini only in anaphase/G1, that then function as a seed for the assembly of the satellite in early G1. The signal that triggers satellite assembly at N-Sfi1 is presently unclear, but may be linked to the kinase Mps1, that not only functions at kinetochores in SAC regulation in yeast, but also in SPB duplication [23,43,44].

## 3. Insertion of the New SPB in *S. cerevisiae*

Studying the duplication cycle and insertion process of the SPB/centrosome is challenging, due to their relative small size and the close vicinity of the parental structure to the daughter. In *S. cerevisiae*, the satellite assembles as described above at the distal end of the bridge. Therefore, the bridge length of 120 nm [36], defines the distance between the old and the new SPB. The resolution limit of a conventional light microscope is approximately 200 nm, as defined by Abbe’s diffraction limit. This restricted the study on the duplication and insertion process of the SPB, for a long time, to EM analysis, which will be discussed in the following section.

A pioneering EM-study by Adams and Kilmartin used synchronized budding yeast cells for a detailed analysis of different stages of the duplication and insertion process. The authors describe a characteristic bend of the NE underneath the inserting DP, and most importantly, they identified a pore-like structure directly next to the DP, which was morphological indistinguishable to an NPC [35]. Furthermore, they speculated that this pore assists membrane fusion for SPB insertion. These observations raised two possibilities. Firstly, the bent NE at the end of the bridge may push the membranes sufficiently together to promote fusion. Secondly, the opening next to the DP is indeed an NPC that directly or indirectly assists in the insertion of the DP into the NE.

**Figure 2:**
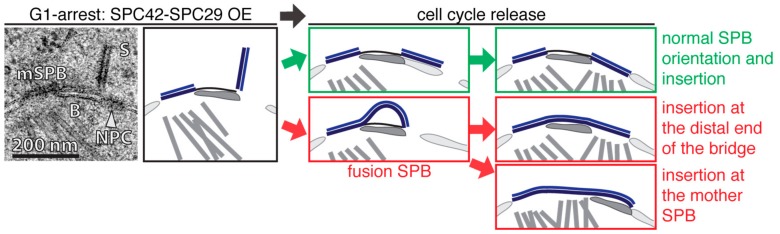
Over-expression (OE) of *SPC42* and *SPC29* in G1 arrested cells. In the top left an EM micrograph and a corresponding comic show a mother SPB (mSPB) of an α-factor arrested cell with an extended satellite (S) after *SPC42* and *SPC29* OE. Both structures are connected via the bridge (B) and a nuclear pore complex (NPC) is localizing adjacent to the S. On the top right (**green**) a normal dSPB orientation and NE insertion after release of the cell cycle block is indicated. In the bottom right (**red**) the formation of a fusion SPB is after the release back into the cell cycle is illustrated. This opens two possibilities for the insertion process. Either the fusion SPB inserts at the distal end of the bridge or proximal via the mSPB opening. For corresponding EM images refer to [40].

Selective over-expression (OE) of two SPB core-components, *SPC42* and *SPC29*, in G1-arrested, satellite bearing cells addressed these possibilities [40]. OE of *SPC42* or *SPC42 SPC29* elongated the satellite from its distal end into a 100–300 nm long and 20 nm wide obelisk-like structure that was oriented nearly orthogonally relative to the NE (Figure 2; left). After α-factor wash out to allow progression into S phase, smaller Spc42-Spc29 obelisks showed a complete and normal NE-insertion, although the central plaque of the dSPB was enlarged (Figure 2, right—green). In cases where the obelisk reached a critical length, the mother and daughter SPBs fused together into one entity called fusion SPBs. However, even this very large SPB inserted into the NE assembled the nuclear plaque and nucleated nuclear MTs. Two different pathways for the NE insertion of the fusion SPB were observed. In some cases the NE insertion process started at the distal end of the bridge, similar to the insertion of a normal dSPB. Other fusion SPBs used the opening provided by the mother SPB to insert into the NE (Figure 2, right—red). However, these data indicate that DP expansion and the insertion into the NE are two independent processes that can be uncoupled from one another. Additionally, the bent NE underneath the DP that was observed by Kilmartin and co-workers [35] was not present in the OE-study [40], and therefore, might indicate that this is not an essential feature for the insertion process of the dSPB into the NE.

**Figure 3:**
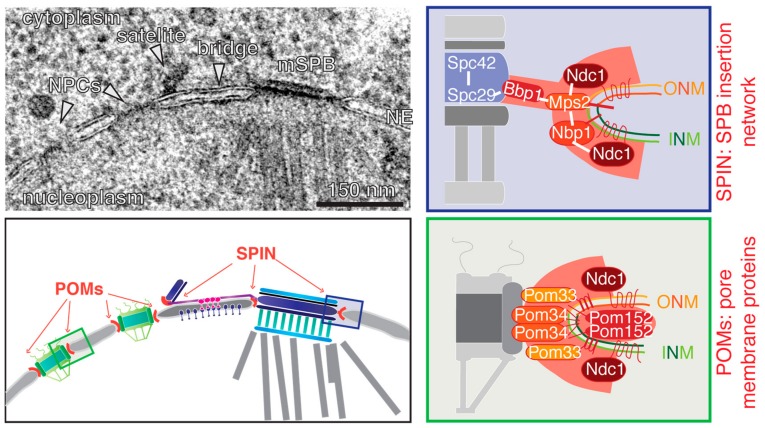
SPB and NPC anchorage complexes. In the top left an EM micrograph is showing a mother SPB (mSPB) and a satellite connected via the bridge. Two NPCs are identified adjacent to the satellite. The corresponding illustration underneath also indicates where the pore membrane proteins (POMs) and SPB insertion network (SPIN) are located; the close-ups on the right list the known components and interactions (**white line**).

The NE is a barrier that physically separates the genetic information in eukaryotes from the cytosol. The double lipid bilayer consists of an outer nuclear membrane (ONM) facing the cytoplasm and an inner nuclear membrane (INM) oriented towards the nucleus. The INM and ONM are separated by a gap, the so-called perinuclear space, which is continuous with the lumen of the endoplasmatic reticulum. This consequently means that the INM and ONM are fused at the NE insertion interface of the SPB, and also the NPC [45,46] (Figure 3). An exhaustive, high-voltage electron tomography study visualized this SPB-membrane interface as a hook-like structure connecting the central plaque with the NE [39]. Specialised proteins, such as members of the SNARE family or viral proteins, usually facilitate membrane fusion [47,48]. However, no such proteins are known to be involved in the SPB or NPC insertion process. This raises the questions of how large proteinaceous structures can become inserted into the NE, and whether SPB NE insertion and NPC biogenesis are linked by common mechanisms, or are even interdependent.

The recent development of super-resolution microscopy techniques, such as photoactivated localisation microscopy (PALM), direct stochastic optical reconstruction microscopy (STORM), and structured illumination microscopy (SIM), and their implementation in yeast [41,49,50], expanded the toolbox for the analysis of SPB-duplication, and gave new insights into this process. It has been shown by SIM and immuno-EM that the pore that was observed by others next to the DP [35,39] is indeed an NPC [40]. This NPC becomes recruited to the SPB insertion side, specifically in G1 phase of the cell cycle. Data based on rapid inactivation of NPCs indicate that this NPC assisted in the insertion of the new dSPB into the NE [40], but it remains unclear what exact function it might have. Before we further discuss this function (see Section 6), we would like to introduce a network of four interacting proteins, Bbp1, Mps2, Nbp1, and Ndc1, that are collectively called the SPB insertion network (SPIN) because of their role in NE insertion of the dSPB, as indicated by the phenotype of conditional lethal mutants. Failure of the function of Bbp1, Mps2, Nbp1, or Ndc1 prevents the insertion of the dSPB into the NE [51,52,53,54,55]. As a consequence of this defect, such cells carry a functional mother SPB, and a “dead” dSPB that is not inserted into the NE, and therefore fails to organize nuclear MTs. The “dead” pole is normally pulled via cytoplasmic MTs/cell cortex interactions into the daughter cell body, while the functional mother SPB together with the replicated DNA stays in the mother cell [42,51].

How can we explain the SPB duplication phenotype of conditional lethal *BBP1*, *MPS2*, *NBP1*, and *NDC1* cells? Except for Bbp1, all SPIN proteins are integral membrane proteins. Bbp1 interacts with Mps2, and this complex is most likely important to dock the membrane interface to the SPB core via the interaction of Bbp1 with the central plaque protein Spc29 [54,56]. Mps2 in turn interacts with the membrane anchor protein Nbp1 [51,54,57]. An N-terminal amphipathic α-helix facilitates the membrane anchoring function of Nbp1 [57]. This α-helix might have a membrane remodelling activity, or could be implemented in sensing membrane curvature [58]. This could be a means to target Nbp1 and interacting proteins to the INM-ONM fusion site that is characterized by a high membrane curvature. Both proteins, Mps2 and Nbp1, interact additionally with the six-transmembrane protein Ndc1, which is a shared component of the SPB and NPC [51,52,54,56] (Figure 3; SPIN). With the aid of large tetraploid SPBs and larger fusion SPB, it was shown by SIM that all four SPIN components encircle the SPB [40,50]. This ring of Bbp1, Ndc1, Nbp1, and Mps2 is flexible, and adapts to the diameter of the SPB. It only assembles during NE insertion of the new SPB. Thus, it is unlikely that it functions as a preformed pore into which the new SPB is inserted. Interestingly, as the fusion SPB described above has an alternative way to insert into the NE via the opening provided by the mother SPB (Figure 2); the observation that these fusion SPBs fell out of the NE in conditional lethal *bbp1*, *nbp1*, *ndc1,* and *mps2* mutant cells strengthens the notion that the SPIN has an SPB anchorage capacity in addition to the proposed insertion function [40].

## 4. Duplication and Insertion of the *S. pombe* SPB

**Figure 4:**
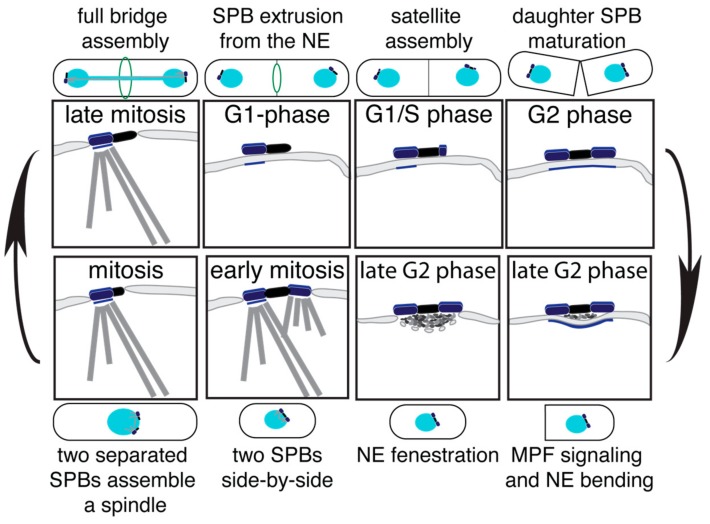
The duplication cycle of the SpSPB. The illustrations indicate the different stages of the SpSPB duplication process, and subsequent nuclear envelope (NE) insertion and ejection. The corresponding cell morphology is indicated additionally.

The duplication cycle of the *S. pombe* SPB (SpSPB) has been studied initially via EM, fluorescence microscopy, and more recently, in a systematic SIM analysis [59,60,61,62]. The SpSPB also has a layered structure that is, however, less pronounced than that of the ScSPB. The SpSPB is inserted into the NE only prior to mitosis [18]. Thus, the duplication process occurs on the cytoplasmic side. Similar to budding yeast, the SPB duplication in *S. pombe* already starts in late mitosis, with elongation of the bridge via the recruitment of Sfi1 [28,63] (Figure 4—late mitosis). Also, the orientation of the Sfi1 molecules are the same as described for *S. cerevisiae*, and the full bridge assembly is likewise mediated via a C-term-to-C-term alignment of antiparallel Sfi1 molecules [62,64]. Interestingly, quantification experiments of the fission yeast Sfi1 molecules revealed a two-step incorporation dynamics. A minor enrichment of approximately 1.5-fold appears in late mitosis, and a second, slower incorporation wave follows from septation until the next mitosis [28,63]. With the aid of a *cdc31* mutant, which blocks the incorporation of Sfi1 and via SIM, it was clarified that the first incorporation wave represents the half bridge to bridge conversion. Later, the bridge expands, most likely to a highly stacked assembly, which is assumed to be important for the proper stabilisation, as fission yeast lacks the bridge anchor protein Kar1 [19,41,62,63]. The new SPB starts to assemble in late G1 [60] (Figure 4—G1/S phase). One of the first proteins to localize to the daughter SpSPB is Ppc89 [62], which is believed to play a similar scaffold function and structural role as Spc42 in the ScSPB [19,65]. With the progression in cell cycle, the satellite matures by the addition of further SpSPB components, and grows in size to a full SPB by G2 phase [60,62] (Figure 4—G2 phase). After septation and commitment to a new cell cycle, the mother and daughter SpSPBs become inserted into the NE. This process starts with the bending of the NE underneath the two SPBs, accompanied by the accumulation of electron-dense material (Figure 4—late G2 phase, right). Next, the so-called polar fenestration, a local disassembly of the NE, allows the duplicated SpSPBs to insert into the NE [18] (Figure 4—late G2 phase, left). Subsequently, both SPBs become activated in order to organize nuclear and cytoplasmic MTs (Figure 4—early mitosis), the bridge structure is severed, and a bipolar spindle forms (Figure 4—mitosis). It has been shown that the phosphorylation of Cdc31 at serine 15 is promoting this bridge separation process. However, this regulation is not absolutely essential for SPB separation [63]. Therefore, further regulatory mechanisms, which have not yet been identified, must be in place. At the end of mitosis, after the full bridge was assembled, the SPB is ejected again from the NE by a process called polar extrusion [18] (Figure 4—G1 phase).

Similar to the insertion process, the extrusion process is also not well understood. However, the Ndc1 homologue Cut11 that also localizes to the SPB and NPC in fission yeast cells shows a specific recruitment to the SpSPB during prophase until mid anaphase [66]. A *cut11* conditional lethal mutant fails to insert the daughter SpSPB into the NE, and displays a tethering problem for the mother SpSPB, which is partially detached from the NE [66]. This phenotype is comparable to the observed “dead” pole and NE extrusion of the fusion SPB observed for *ndc1* mutants in *S. cerevisiae* [40,52]. A very similar phenotype has been reported for *cut12.1* conditional mutants [67]. As this phenotype was further analysed, it turned out that Cut12 works rather upstream of Cut11 as a functional control element to “activate” the SPB for the insertion process [68,69].

One of the first identified SpSPB components was the transmembrane protein Sad1, the homologue of the *S. cerevisiae* Mps3 [70]. The SUN-domain protein Sad1 localizes to the INM underneath the SpSPB [62,70], where it most likely helps to tether the SpSPB to the NE via the interaction with the KASH-domain proteins Kms1 and Kms2 [71,72]. A recent study showed how Sad1 relocalizes during the onset of mitosis to a ring-like structure encircling the SPB, and therefore, implements an insertion or anchorage function [62]. Additionally, Sad1 is a known component of the conserved linker of nucleoskeleton and cytoskeleton (LINC) complex, and connects the cytoplasmic SPB to the centromeres in interphase or telomeres during meiosis [73,74,75]. This interaction is important to ensure SPB insertion during meiotic cell divisions [76]. A third functional player for the SpSPB insertion process is the transmembrane protein Brr6, which harbours four highly conserved cysteine residues in between the two transmembrane regions. Brr6 is recruited to the SpSPB specifically during the NE insertion and extrusion process. Generation of the temperature sensitive mutant *brr6.ts8* reveals an SpSPB insertion defect for the new and “inactive” daughter SpSPB. It was therefore concluded that Brr6 is important to generate the polar fenestration [77]. As there are no definite homologues for the other *S. cerevisiae* SPIN components Npb1, Bbp1, and Mps2, it is not clear if Cut11 is sufficient to fulfil the SpSPB anchorage function on its own, together with Sad1 or if further, non-described SpSPB components play a role here.

## 5. The Nuclear Pore Complex (NPC)

Just like SPBs, NPCs also span the double membrane of the NE. NPCs promote regulated shuttling of molecules between the cytoplasm and the nucleus. The NPC is a large, multi-protein assembly forming a channel with an internal 8-fold symmetry [78,79]. The NPC is built of ~30 different nucleoporins (Nups) that are present in 8, 16 or 32 copies per NPC [80,81]. At the interface of NPC and NE, where the INM and ONM are fused together, the NPC is anchored to the NE via integral membrane proteins, or so-called pore membrane proteins (POMs). Ndc1, the shared component between NPC and SPB, is one of these POMs, and together with Pom33, Pom34, and Pom152, it forms the membrane interface to embed the NPC into the NE [82,83,84,85,86] (Figure 3, POMs).

**Figure 5:**
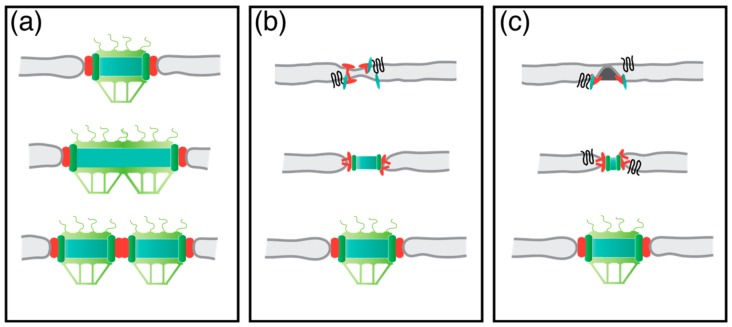
Different models of NPC biogenesis. Model in (**a**) shows the growth of one NPC, which will later split into two. Model (**b**) demonstrates the simultaneous deformation of the NE from the in- and outside. Model (**c**) illustrates the inside-out assembly of a NPC. The POM-anchorage complex is illustrated in red; green, nucleoporins (Nup) components; black, membrane shaping proteins.

In higher eukaryotic cells with NE-breakdown in mitosis, NPCs can be assembled via two different pathways. One is the reassembly at the end of mitosis, together with the reassembly of the NE. A second one is the de-novo NPC biogenesis during interphase. This requires membrane fusion of the INM with the ONM. As yeast has a closed mitosis, yeast cells are limited to the second pathway for the assembly of new NPCs. For some time, there were two main models trying to explain the NPC assembly into a closed membrane sheet. Firstly, the NPC grows in size and later splits into two (Figure 5a). The observation of NPCs with more than the 8-fold symmetry could support this model [87]. Secondly, a new NPC assembles on the NE from inside and outside. This brings both membranes close together, and ultimately results in membrane fusion and full NPC assembly [88] (Figure 5b). Until recently, there were no experimental data which would produce distinctions between both models. However, a new study in HeLa-cells that correlated live cell microscopy and EM points to a third possibility of NPC biogenesis—the inside-out assembly. This study describes how an 8-fold symmetric ring-like structure assembles on the INM, and thereby deforms the INM into a mushroom-like structure. This mushroom structure then grows until the bent INM fuses with the ONM, and allows the full assembly of a new NPC [89] (Figure 5c). Further evidence to support this inside-out NPC assembly model comes from a recent study in budding yeast. Here, it was described that the two membrane proteins, and orthologues Brl1 and Brr6, are localizing to the assembly site of new NPCs, where they seem to be important for the fusion of the INM and ONM [90].

## 6. Closing Remarks and Future Directions

The common feature of all eukaryotic organisms is the compartmentalization of the cell. Thereby different organelles are defined to create and ensure the microenvironment for certain biological processes. One of these compartments is the nucleus which is enclosed by the NE. NPCs and SPBs are two macro-molecular complexes embedded in the double lipid bilayer, to enable the shuttling of molecules across the NE and organize spindle microtubules, respectively. As described in this review, there is growing evidence that both complexes are intertwined more than one might expect at first sight. One key finding is the functional role of NPCs during the ScSPB duplication and NE insertion process. However, the exact functional relevance and precise function of the observed NPC in vicinity to the inserting daughter ScSPB needs to be determined. One possibility is the delivery of SPB components. An obvious candidate is Ndc1, which is a shared anchorage factor between both complexes. Another function could be the allocation of the membrane fusion side to directly insert the ScSPB into the NE via the NPC. In this respect, the question of what exactly happens to the NPC during and after the insertion process should be investigated. Is the NPC disassembled at any point or released sideways, therefore, staying intact? Furthermore, what is the physical link or recruitment factor of the NPC to the duplicating ScSPB? Interestingly, Spc29, one of the first components localizing to the new ScSPB, was originally described as the nuclear import protein (Nip29), and several links between Spc29 and the NPC have been made [91,92]. Conversely, the filamentous NPC basket protein Mlp2 has been shown to strongly interact with the ScSPB via Spc42, Spc29, and Spc110 [93], but since Mlp2 is not an essential gene, it cannot be the only physical link between ScSPB and NPC. However, there are further surprising genetic interaction between ScSPB and NPC components which connect both complexes. The SPIN component *MPS2* is an essential gene, but the deletion can be rescued in a *∆mlp1/∆mlp2* or *∆pom* (e.g., *POM152* or *POM34*) strain background [94]. The same applies for the gene coding for the ScSPB bridge and transmembrane protein Mps3 [95]. It has been speculated that this rescue of *MPS2* or *MPS3* is caused by a change in membrane rigidity, or an overall higher availability of Ndc1 [45]. However, the fact that additional gene dosage of *NDC1* does not rescue *mps2∆* lethality casts doubt upon this interpretation.

Brr6 is one important insertion factor for the SpSPB [77]. In budding yeast, Brr6 is present, together with its homologue Brl1, which arose most likely from a gene duplication [96]. Both proteins have been shown to be important for NPC biogenesis, but seem to have no prominent role in SPB duplication [90]. It should be tested, whether this is a functional variance of Brr6 in different species, or if the effect on NPC formation in *S. pombe* has simply not yet been identified.

Taken together, we predict that a deeper understanding of the SPB duplication and insertion process in yeast model organisms will also improve our knowledge of NPC biogenesis, which is a universal feature of all eukaryotic cells.
